# Predictors of mortality among bacteremic patients with septic shock receiving appropriate antimicrobial therapy

**DOI:** 10.1186/1471-2253-14-21

**Published:** 2014-03-25

**Authors:** David D Leedahl, Heather A Personett, Ognjen Gajic, Rahul Kashyap, Garrett E Schramm

**Affiliations:** 1 Pharmacy Services (DDL), Sanford Medical Center, 801 Broadway, Fargo, ND 58122, USA; 2 Pharmacy Services (HAP, GES), and Division of Pulmonary and Critical Care Medicine (OG and RK), Mayo Clinic, 200 First St SW, Rochester, MN 55905, USA

**Keywords:** Septic shock, Bacteremia, Antimicrobials, Outcome, Hospital mortality

## Abstract

**Background:**

Factors capable of impacting hospital mortality in patients with septic shock remain uncertain. Our objective was to identify predictors of hospital mortality among patients who received appropriate antimicrobial therapy for bacteremic septic shock after accounting for severity of illness, resuscitation status, and processes of care.

**Methods:**

We conducted a secondary subgroup analysis of a prospective severe sepsis cohort study. Patients with septic shock and positive blood cultures who received appropriate antimicrobial therapy were included. Univariable analyses were used to identify differences between hospital survivors and non-survivors, and a multivariable logistic regression model revealed independent determinants of hospital mortality.

**Results:**

From January 2008 to December 2010, 58 of 224 included patients died in the hospital. Multivariable logistic regression analysis demonstrated 2 independent predictors of hospital mortality. These included continuous renal replacement therapy utilization within 48 hours of septic shock recognition (adjusted odds ratio [OR], 5.52; 95% confidence interval [CI], 1.94-16.34) and intra-abdominal infection (adjusted OR, 3.92; 95% CI, 1.47-10.79). *Escherichia coli* was independently associated with a lower risk of hospital mortality (adjusted OR, 0.34; 95% CI, 0.11-0.90).

**Conclusion:**

Intra-abdominal infection and continuous renal replacement therapy were associated with increased hospital mortality in patients with septic shock who received appropriate antimicrobial therapy. Our findings may be explained by suboptimal intra-abdominal infection management or inadequate antimicrobial concentration in these patients.

## Background

Severe sepsis and septic shock account for up to 10% of intensive care unit (ICU) admissions, and the incidence of this syndrome is anticipated to reach 1.1 million cases annually in the United States by 2020 [1,2]. Scripted treatment guidelines from the Surviving Sepsis Campaign [3] and the Institute for Healthcare Improvement [4] have focused on a bundled approach to care, yet global compliance remains poor [5]. Within the septic shock treatment armamentarium, a directed approach that includes both appropriate and timely antimicrobial therapy continues to be a cornerstone of treatment, with ample evidence supporting an association between inappropriate or delayed prescription of antimicrobials and increased patient mortality [6-9].

Despite advances in recognition and treatment of septic shock, hospital mortality remains alarmingly high, ranging from 30% to over 50% in recent publications [5,10,11]. Documented bacteremia has been proposed as the fundamental pathophysiological determinant of sepsis, and although not a requirement for diagnosis, bacteremia should be established in septic patients when possible [10,12,13]. Outcomes in bacteremic sepsis have been influenced by age, sex, severity of illness, albumin, comorbidities, pathogen, and source. Although prompt administration of appropriate antimicrobial therapy may improve hospital survival, predictors of mortality in bacteremic septic shock remain largely unknown when appropriate antimicrobials are administered [9]. Thus, we hypothesized that independent risk factors for hospital mortality exist in bacteremic patients with septic shock who received appropriate antimicrobial therapy while accounting for Acute Physiology and Chronic Health Evaluation (APACHE) III score, preference of care, and processes of care, including compliance with non-antimicrobial elements of our sepsis resuscitation bundle.

## Methods

In a secondary subgroup analysis of a prospective observational trial, we evaluated 1304 patients with septic shock to identify risk factors for hospital mortality and further improve the delivery of care at our institution. Data were from patients admitted to a 24-bed medical ICU of Mayo Clinic, Rochester, MN, from January 2008 through December 2010. The study was approved by the Mayo Clinic Institutional Review Board (IRB# 11-004905) and therefore was performed in accordance with the ethical standards laid down in the 1964 Declaration of Helsinki and its later amendments. Informed authorization was obtained from patients for their involvement in the original prospective study. The Mayo Clinic IRB waived the requirement for informed consent for our study, and all included patients authorized their medical records to be reviewed for research. Our original prospective study included patients with suspected infection, age ≥18 years, and with systolic blood pressure <90 mm Hg, despite a fluid challenge of 20 ml/kg body weight of crystalloid or lactate level >4 mmol/L. Patients who refused septic shock resuscitation (including placement of a central catheter), or experienced active bleeding or cardiogenic pulmonary edema were excluded [14]. Our secondary cohort analysis included patients who had microbiologically confirmed bacteremia upon initial blood cultures, received appropriate antimicrobial therapy within 24 hours and met consensus criteria for septic shock. All patients included in the study timeframe were admitted in our medical ICU after implementation of daily auditing and weekly feedback regarding sepsis resuscitation bundle compliance [14]. Only the index episode of septic shock was included for patients with a recurrent episode within the study timeframe. The study ICU was staffed by a multidisciplinary team lead by intensivists, with 24/7 in-house coverage [15].

### Data elements

The following information was collected for each patient: demographics (age, gender, race), ICU admission source, APACHE III score, hospital length of stay, compliance with individual sepsis bundle elements, antimicrobials administered, renal replacement modalities utilized, do not resuscitate (DNR) status in case of cardiac arrest at time of septic shock recognition, initial plasma lactate level, and hospital mortality. An additional file provides a description of our sepsis bundle elements in more detail (see Additional file 1). Severity of illness was assessed using APACHE III scores, which were calculated using technology at our institution as described previously [16]. We recorded pathogens from all microbiology cultures and antimicrobials administered within 24 hours of blood culture or septic shock recognition, whichever occurred first.

### Definitions

Septic shock was defined per the American College of Chest Physicians and Society of Critical Care Medicine consensus criteria [17], with the definition of bacteremia being consistent with International Sepsis Forum Consensus Conference recommendations [12]. The time of septic shock recognition was defined as the time of documented hypotension despite 20 ml/kg of crystalloid. For patients transferred from outside hospitals to our institution with septic shock, the ICU admission time was considered the time of recognition. Appropriate antimicrobial therapy was defined as antimicrobial agent(s) administered within 24 hours of septic shock recognition to which pathogen(s) that were subsequently isolated and identified from all available microbiology cultures (in addition to blood cultures) had documented in vitro susceptibility, consistent with other investigations [6,7,11]. Preference of care was defined as the presence of “DNR in case of cardiac arrest” status in the electronic medical record at the time of septic shock recognition, and was collected due to previous investigations regarding the impact of preference of care on post-ICU mortality [18]. During the study timeframe, a sepsis response team (SRT) was implemented in our ICU to improve the process of patient care [14]. Decisions regarding the choice and duration of antimicrobial agents were at the discretion of treating physicians and made in collaboration with clinical pharmacists. The source of bacteremia was documented in the electronic medical record and was classified as either primary (catheter, implantable cardioverter-defibrillator, pacemaker, arteriovenous fistula, ileal conduit, or endocarditis related) or secondary. If a secondary source of infection was suspected, the infection source was designated as skin/soft tissue, respiratory, urinary, intra-abdominal (IA), and others. When a secondary source of infection was not documented, the source was classified as unknown. *Staphylococcus epidermidis* was not considered pathogenic as described previously [8]. *Candida* species from blood were considered pathogenic, but *Candida* species isolated from bronchioalveolar lavage, sputum, tracheal secretion, and urine without documented candidemia were considered colonizers. Stress dose steroid administration was defined as receiving ≥ 50 mg of intravenous hydrocortisone during the first 24 hours after septic shock recognition. *Clostridium* species isolated from stool were considered universally susceptible to oral and intravenous metronidazole or oral vancomycin. *Clostridium difficile* related diseases were considered an IA source of bacteremia. Source control of IA infection during hospital admission was defined as any abdominal drainage of infected foci or a surgical procedure to control IA contamination in an attempt to restore anatomic and physiological integrity, according to Infectious Diseases Society of America guidelines [19].

### Continuous renal replacement therapy (CRRT)

The CRRT modality of choice for adults at our institution is continuous veno-venous hemofiltration (CVVH), which removes solutes by convection. All CVVH treatments applied the Prismaflex System and the HF 1400 polyarylethersulfone filter (Gambro, Stockholm, Sweden). The standard blood flow rate was 200 ml/min, with sodium citrate anticoagulation. Prismasate (Gambro, Inc., Lakewood, CO) replaced the hemofiltration fluid, customarily administered 50% prefilter and 50% postfilter. No formal criteria existed for CVVH initiation during the study period. Use of CVVH was per intensivist and attending nephrologist discretion and individualized for each patient.

### Antimicrobial susceptibility

Our microbiology laboratory services performed antimicrobial susceptibility of isolates using a non-automated agar dilution method according to breakpoints established by the Clinical and Laboratory Standards Institute and in publication between 2008 and 2010 [20-22]. Susceptibility profiles of isolates were based on *in vitro* analysis using established breakpoints. The classification of intermediate-resistance *in vitro* was considered resistant for our study.

### Statistical analysis

Categorical variables were summarized as frequency (%) and compared between hospital survivors and non-survivors using chi squared tests. Continuous variables were expressed as mean ± standard deviation or median with interquartile range (IQR) as appropriate. Continuous variables were compared using t-tests for parametric data or Wilcoxon analysis for nonparametric data. It was determined that 175 patients would provide 80% power to detect a two-fold increased risk of hospital mortality for a given variable, assuming 40% nonsurvivors and an alpha level of 0.05. Baseline, epidemiologic, and treatment variables were initially assessed by univariable analyses. Variables associated with and clinically relevant to the dependent outcome of increased hospital mortality with a p-value ≤ 0.1 were included in multivariable analysis. In the event that two related variables were determined to have a p-value <0.1 (such as gram positive infection and *Staphylococcus aureus* infection), the more specific variable was included in multivariable analysis. To promote a reliable multivariable model, the authors decided *a priori* to adjust the multivariable model for the following variables: APACHE III score (severity of illness), DNR in case of cardiac arrest status (preference of care), enrollment after implementation of a SRT in the study ICU, timing of appropriate antimicrobial therapy, and compliance with non-antimicrobial elements of the sepsis resuscitation bundle (processes of care). Only cumulative mortality rates were estimated using Kaplan-Meier methodology to describe mortality at week 1 and 2 after septic shock recognition. Multivariable logistic regression analysis was performed to identify independent predictors of hospital mortality. A p-value of <0.05 was considered statistically significant in multivariable analysis. Data were analyzed using JMP software, version 9.0.1 (Cary, NC).

## Results

**Figure 1:**
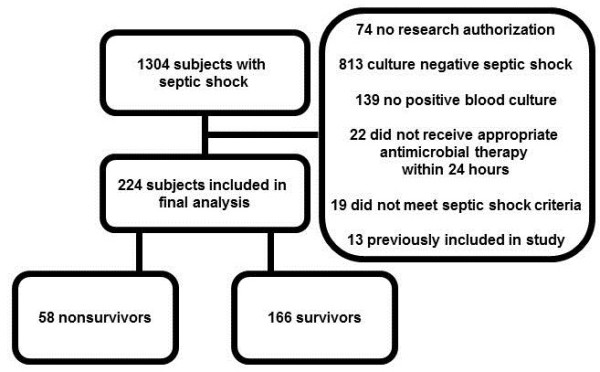
Cohort development.

Of the 1304 episodes of septic shock evaluated, 491 patients had at least one positive microbiologic culture and 224 met inclusion and exclusion criteria (Figure 1). APACHE III score was the only baseline characteristic statistically associated with hospital mortality by univariable analysis (Table 1). Fifty-eight of 224 patients died in the hospital, and the cumulative mortality rates within 1 and 2 weeks were 12.1%, 18.75%, respectively. The maximum observed length of stay was 164 days, whereby the cumulative mortality rate was 39.2%. The median length of stay was 8 days, and the median time to death for patients who died in the hospital was 6.5 days (IQR 2-15 days). Epidemiologic and clinical characteristics of the bacteremic infections are summarized in Table 2.

**Table 1:** Baseline characteristics

	**Survivors**	**Non-survivors**	**P**
	**n = 166**	**n = 58**	
Caucasian (%)	155 (93.4)	53 (91.4)	0.612
Male gender (%)	98 (59.0)	30 (51.7)	0.333
Age, years, median (IQR)	72 (58-81)	72 (63-80)	0.534
APACHE III score, median (IQR)	58 (47-72)	67 (53-80)	0.014
Admission from emergency department (%)	101 (60.8)	35 (60.3)	0.947
Transfer from outside facility (%)	25 (15.4)	6 (10.3)	0.371
Do not resuscitate in case of cardiac arrest (%)	36 (21.7)	15 (25.9)	0.514
Lactate, mmol/L, median (IQR)	2.6 (1.7-3.9)	3.02 (2.0-4.7)	0.151
Enrolled after sepsis response team implementation at study ICU (%)	119 (71.7)	40 (69.0)	0.849

*APACHE*, Acute Physiology and Chronic Health Evaluation score; *SD*, standard deviation; *IQR*, interquartile range.

**Table 2:** Epidemiologic and clinical characteristics of the 224 included patients and their relationship to hospital mortality

**Variable**	**Survivors n (%)**	**Non-survivors n (%)**	**P**
	**n = 166**	**n = 58**	
IHD within 48 hours	9 (5.4)	5 (8.6)	0.386
CRRT within 48 hours	11 (6.6)	15 (25.9)	<0.01
Stress dose steroid	61 (36.8)	29 (50.0)	0.076
Gram stain of organisms isolated from all cultures			
Fungi or parasite	4 (2.4)	1 (1.7)	0.761
Gram positive	48 (28.9)	26 (44.8)	0.027
Gram negative	80 (48.2)	21 (36.2)	0.114
Polymicrobial	34 (20.5)	10 (17.2)	0.593
Concomitant positive culture (when culture results available)			
Respiratory	17 (13.4)	8 (18.6)	0.404
Urine	53 (34.4)	10 (20.4)	0.065
Primary bacteremia	16 (9.6)	9 (15.5)	0.221
Source of infection if secondary bacteremia^a,b^			
Skin soft tissue	20 (12.1)	6 (10.3)	0.727
Respiratory	30 (18.1)	11 (19.0)	0.880
Urinary	57 (34.3)	9 (15.5)	0.007
Intra-abdominal	20 (12.1)	16 (27.6)	0.006
Unknown	22 (13.3)	5 (8.6)	0.351
Source control procedure if intra-abdominal source^c^	16 (80.0)	13 (81.3)	0.925
Median time to source control, hours (IQR)^c^	13.5 (0.7-65.0)	13.9 (4.9-51.4)	0.568
Vasopressor for at least 1 hour	96 (57.8)	43 (74.1)	0.028
Antimicrobials administered^d,e^			
Carbapenem (not ertapenem)	48 (28.9)	11 (19.0)	0.139
Cefepime	33 (19.9)	17 (29.3)	0.138
Ceftriaxone	20 (12.1)	10 (17.2)	0.318
Clindamycin	9 (5.4)	4 (6.9)	0.679
Fluoroquinolone	130 (78.3)	43 (74.4)	0.514
Levofloxacin	103 (62.1)	32 (55.2)	0.357
Metronidazole	12 (7.2)	11 (19.0)	0.011
Piperacillin/tazobactam	89 (53.6)	28 (48.3)	0.484
Vancomycin	142 (85.5)	47 (81.0)	0.416
Antifungal agent	4 (2.4)	2 (3.5)	0.673
Appropriate antimicrobial therapy administered <1 hr	128 (77.1)	41 (70.7)	0.328
Time to appropriate antimicrobial therapy, hours, median (IQR)	0 (0-0.87)	0.23 (0-1.08)	0.033

*IHD*, intermittent hemodialysis; *CRRT*, continuous renal replacement therapy; *IQR*, interquartile range. ^a^n = 150 survivors, 49 non-survivors; ^b^1 case of each (not included in analysis): periodontal, ear, kidney stone; ^c^n = 36; ^d^Due to administration of ≥ 1 antimicrobial per patient, total n is >224; ^e^Antimicrobials with <5 administrations not included in analysis.

The impact of each antimicrobial on hospital mortality was analyzed independently during univariable analyses (Table 2). Vancomycin, fluoroquinolones, and piperacillin/tazobactam were the most frequently prescribed antimicrobials, administered to 84%, 77%, and 52% of patients, respectively. Patients receiving appropriate antimicrobial therapy within 1 hour were evenly distributed between hospital survivors and non-survivors (p = 0.328). The median time to appropriate antimicrobial therapy for the entire cohort was 0 hours (IQR 0-1.0, minimum 0 hours, maximum 23.0 hours), but the median time to appropriate antimicrobial therapy was statistically different between groups (Table 2). Metronidazole was the only antimicrobial agent associated with increased hospital mortality upon univariable analysis (Table 2). Secondary analyses revealed that patients who received metronidazole had no statistically significant differences in the following variables when compared to those not receiving metronidazole: age, baseline serum lactate, IA source of infection, source control procedures for IA infection, utilization of CRRT within 48 hours, APACHE III score, or requirement of vasopressors (analysis reports not shown).

Gram positive infections were associated with a higher risk of hospital mortality by univariable analysis (Table 2). *Escherichia coli* was the only pathogen statistically more common in survivors (Table 3). Patients with septic shock related to *E. coli* infection were not statistically younger, nor were their median APACHE III score or baseline lactate lower compared to patients with non-*E. coli* infections.

**Table 3:** **Microorganisms isolated from blood**^**a**^

**Variable**	**Survivors n (%)**	**Non-survivors n (%)**	**P**
	**n = 166**	**n = 58**	
Gram negative aerobes	99 (59.6)	26 (44.8)	0.051
E coli	51 (30.7)	7 (12.1)	0.005
Klebsiella spp	23 (13.9)	9 (15.5)	0.756
Pseudomonas aeruginosa	8 (4.8)	4 (6.9)	0.545
Other gram negative	22 (13.3)	8 (13.8)	0.917
Gram positive aerobes	66 (39.8)	33 (56.9)	0.024
Staphylococcus aureus	27 (16.3)	16 (27.6)	0.060
Methicillin susceptible	13 (7.8)	8 (13.8)	0.180
Methicillin resistant	14 (8.4)	8 (13.8)	0.238
Streptococcus pneumoniae	12 (7.2)	5 (8.6)	0.730
Enterococcus spp	14 (8.4)	7 (12.1)	0.414
Other gram positive	14 (8.4)	5 (8.6)	0.968
Anaerobes	5 (3.0)	2 (3.5)	0.869
Fungi	4 (2.4)	1 (1.7)	0.761

^a^Due to ≥ 1 blood isolate per patient, total n is >224.

Urinary source of infection was associated with increased hospital survival, while IA source of infection was predictive of increased hospital mortality despite similar rates of source control procedures for IA source of infection between groups (Table 2). For patients with IA source of infection, the median time to source control was 13.7 hours (IQR 3.9-54.5 hours) and did not differ significantly between hospital survivors and nonsurvivors (Table 2). During the study period, 55% of patients had full compliance with non-antimicrobial elements of the sepsis resuscitation bundle. Compliance with individual sepsis bundle elements was not statistically different between hospital survivors and non-survivors (Table 4).

**Table 4:** The relationship between mortality and compliance with non-antimicrobial sepsis bundle elements

**Sepsis bundle element**	**Survivors (n = 166)**	**Non-survivors (n = 58)**	**P**
Lactate measured	163 (98.2)	57 (98.3)	0.967
Blood culture before antibiotics	163 (98.2)	58 (100)	0.303
Adequate fluid	127 (76.5)	45 (77.6)	0.867
Appropriate vasopressor	144 (86.8)	52 (89.7)	0.564
Appropriate red blood cell transfusion	150 (90.4)	51 (87.9)	0.600
Appropriate inotrope use	103 (62.1)	37 (63.8)	0.813
Full adherence to non-antimicrobial elements of the sepsis bundle	93 (56.0)	31 (53.5)	0.734

Four variables with a p-value of <0.1 by univariable analysis were not included in multivariable analysis (gram positive infection, gram negative infection, gram positive aerobe isolate, gram negative isolate) due to a more specific, related variable in the multivariable model (*Staphylococcus aureus* and *E. coli* infection). Utilization of CRRT within 48 hours and IA source of infection were independently associated with increased hospital mortality by multivariable logistic regression analysis, and septic shock related to *E. coli* infection was independently associated with a lower risk of hospital mortality (Table 5). An interaction between *E. coli* infection and urinary source of infection was not observed. A sensitivity analysis was also conducted whereby patients transferred from an outside facility were excluded, yielding similar results (Additional file 2).

**Table 5:** Multivariable logistic regression analysis to determine independent predictors of hospital mortality

**Variable**	**Adjusted odds ratio**	**95% confidence interval**	**P**
Continuous renal replacement therapy within 48 hours	5.52	1.94-16.34	0.001
Intra-abdominal source of infection	3.92	1.47-10.79	0.006
*Escherichia coli* infection	0.34	0.11-0.90	0.029
APACHE III score, per unit increase	0.99	0.98-1.01	0.323
Vasopressor for at least 1 hour	1.77	0.77-4.21	0.181
Lactate, per 1 mmol/L increase	0.98	0.84-1.13	0.736
Do not resuscitate in case of cardiac arrest	1.95	0.86-4.40	0.109
Full adherence to non-antimicrobial elements of the sepsis bundle	0.83	0.38-1.81	0.646
Median time to appropriate antimicrobial therapy, per hour delay	0.98	0.87-1.15	0.769
Enrolled after sepsis response team implementation at study ICU	1.21	0.57-2.72	0.618
Urinary source of infection	0.72	0.26-1.88	0.506
*Staphylococcus aureus* infection	2.34	0.95-5.83	0.064
Received metronidazole	2.79	0.99-7.88	0.053

*APACHE*, Acute Physiology and Chronic Health Evaluation III score; *ICU*, intensive care unit.

## Discussion

Our study offers additional perspective to sepsis mortality literature, which has customarily reported poor patient outcomes due to delayed or inappropriate antimicrobial administration [6-9]. The current body of literature addressing predictors of septic shock mortality is remarkably limited to date, especially in the setting of appropriate antimicrobial therapy, bundled sepsis treatment, and a dedicated SRT. We highlight CRRT and IA source of infection as independent risk factors for hospital mortality after adjusting for severity of illness, preference of care, and processes of care including compliance with non-antimicrobial elements of our sepsis resuscitation bundle.

Labelle and colleagues recently conducted a retrospective study of bacteremic septic shock patients receiving appropriate initial antibiotic treatment, identifying APACHE II score and ICU-acquired infection as the most important determinants of hospital mortality [11]. Alternatively, septic shock caused by methicillin-susceptible *Staphylococcus aureus* (MSSA) infection was independent predictor of survival, and the investigators subsequently concluded MSSA infection was a surrogate for lower severity of illness and younger age. In our cohort, *E coli* infection was independently associated with hospital survival, and these patients did not differ from those with non-*E. coli* infections in regard to age, APACHE III score, or baseline serum lactate. Our study supplements the work of Labelle and colleagues by evaluating additional treatment variables in a more recent patient cohort enrolled between 2008 and 2010. However, patients in our study were not identified by the presence of International Classification of Disease codes, but rather by achieving clinical criteria for septic shock. In addition, our patients received uniform treatment guided by a sepsis resuscitation bundle during the implementation of a SRT.

Adjunctive therapies, such as CRRT, are frequently used to treat critically ill patients with multiple organ failure. Small retrospective studies have suggested that early initiation of CRRT may improve clinical outcomes in septic AKI [23]. However, no consensus exists for the optimal timing of CRRT in patients with septic shock and AKI [24], and early CRRT has also been associated with worse outcomes [25]. Our finding of increased mortality when CRRT is initiated within 48 hours of septic shock encourages further investigation into the appropriate timing of CRRT in the setting of severe sepsis and septic shock.

Utilization of CRRT is indicative of organ dysfunction, a well-known contributor to sepsis related mortality and may explain the associated mortality in our study [26]. Additional markers of renal dysfunction were not included in our analysis as APACHE III accounts for serum creatinine, urine output, blood urea nitrogen, and the presence of acute renal failure. While CRRT may provide benefit in sepsis [27], CRRT may also clear therapeutic agents from the body and alter drug pharmacokinetics, potentially leading to inadequate drug response or treatment failure [28]. The CRRT modality of choice for adults at our institution is CVVH, which removes solutes by convection. Although our multivariable model was adjusted for APACHE III score, we can not preclude the possibility that CRRT was a surrogate for severity of illness or potentially inadequate antimicrobial tissue concentrations. Of note, our institution has a standardized antimicrobial dosing algorithm for patients on CRRT, which is provided in an additional file (see Additional file 3).

Intra-abdominal infections are the second most common cause of septic death in the ICU [29]. Our findings are similar to those of Labelle and colleagues, who found IA source of gram-positive bacteremia to be predictive of hospital mortality in septic shock patients [11]. Additionally, our analysis demonstrated that IA source of septic shock was predictive of mortality regardless of pathogen(s) isolated, time to procedural source control, or whether procedural source control was performed. Our findings highlight the importance of early identification and assessment and management of patients presenting with a suspected IA source of septic shock.

Strengths of our study include that it is the first to investigate additional modifiable predictors of septic shock mortality (eg. antimicrobials, renal replacement modalities, sepsis bundle elements) in the setting of appropriate antimicrobial therapy. In addition, we used the time of documented hypotension, rather than vasopressor initiation, to identify the time of septic shock recognition. We also excluded patients without documented bacteremia in order to derive a septic shock cohort wherein the causative organism was best defined. Several limitations of our study must also be noted. Our definition of appropriate antimicrobial therapy deviates from the current recommendations from the Surviving Sepsis Campaign and the Institute for Healthcare Improvement. Our criterion was established to aid in evaluation of possible mortality predictors in the broader, clinical picture of resuscitation and management of severe sepsis and septic shock, similar to the methodology of two recent sepsis-related mortality investigations [9,11]. Since our study was conducted at a single ICU with bacteremic patients who were predominantly Caucasian, the findings may not be generalizable to patients with different baseline characteristics in varying practice environments or those without positive blood cultures. Similar to other studies of this nature, we were limited to recording septic shock recognition as the time of ICU admission for patients transferred from outside hospitals. Individual antimicrobials are frequently part of a multiple antimicrobial regimen in severe sepsis and septic shock, therefore, statistical analysis of antimicrobials independently does not emulate typical prescribing practices. It is important to recognize that our findings mainly address the association of various factors with hospital mortality after septic shock and not necessarily sepsis-related mortality. Athough all patients receiving CRRT had a primary renal indication (e.g. oliguria or anuria, hyperkalemia, fluid overload unresponsive to diuresis), we cannot exclude the possibility that undetected or undocumented differences in the criteria to start CRRT in our study may have influenced the results. The relatively large number of data elements analyzed at the univariable level, like other studies of this nature, may yield a lack of power to detect associations of interest between hospital mortality and single variables. We must note that the statistically significant variables identified in the multiple logistic regression model demonstrate unique information about hospital mortality within each variable. Moreover, our analysis revealed an association with hospital mortality beyond that which may be explained by related variables (CRRT and APACHE III score). Despite the establishment of appropriate antimicrobial therapy by *in vitro* susceptibility to antimicrobials administered, we were not able to address the issue of antibiotic adequacy (eg. dosing, therapeutic drug levels, drug penetration). Our definition of appropriate antimicrobial therapy required documented susceptibility of all subsequently isolated microorganisms to antimicrobials administered, regardless of sensitivity profile, and limits the impact of multidrug resistance on our findings.

## Conclusions

In conclusion, the use of CRRT within 48 hours of septic shock recognition and IA source of infection were independent predictors of hospital mortality after adjustment for severity of illness, preference of care, and processes of care including compliance with non-antimicrobial elements of our sepsis resuscitation bundle. Our findings pertaining to IA infection and CRRT are hypothesis generating and may be explained by suboptimal IA infection management or inadequate antimicrobial concentration in these patients.

### Key messages

•Mortality after septic shock remains high despite advances in recognition and treatment.

•When appropriate antimicrobial therapy is administered, CRRT within 48 hours and intra-abdominal source of infection were associated with increased hospital mortality after accounting for preference and processes of care.

•Clinical studies and quality improvement interventions should focus on the role of intra-abdominal infection management and the utility and timing of CRRT in these patients.

## Abbreviations

APACHE: Acute physiology and chronic health evaluation; CI: Confidence interval; CRRT: Continuous renal replacement therapy; CVVH: Continuous venovenous hemofiltration; IA: Intra-abdominal; ICU: Intensive care unit; IRB: Institutional review board; IQR: Interquartile range; SRT: Sepsis response team.

## Competing interests

No financial support was required to perform the study. The authors declare no financial or non-financial conflicts of interest.

## Authors’ contributions

DL participated in data acquisition and study design, performed statistical analyses, and drafted the manuscript. HP participated in study design, critically revised the manuscript, and helped with interpretation of the data. OG made substantial contributions in the conception and design of the study, helped with data interpretation, and revised the manuscript for important intellectual content. RK participated in data acquisition and helped with statistical analysis, data interpretation, and manuscript revisions. GS conceived and coordinated the study, acquired data, helped with interpretation of the data, and helped draft the manuscript. All authors read and approved the final version of the manuscript for publication.

## Pre-publication history

The pre-publication history for this paper can be accessed here:

http://www.biomedcentral.com/1471-2253/14/21/prepub

## Supplementary Material

Additional file 1 **Elements of the sepsis resuscitation bundle.** Detailed explanation of the sepsis resuscitation bundle at our institution. Click here for file

Additional file 2 **Independent predictors of hospital mortality by logistic regression analysis after excluding patients transferred from an outside facility.** A sensitivity analysis of the multivariable analysis whereby patients transferred from an outside facility were excluded, yielding similar results. Click here for file

Additional file 3 **Adult Dosing for Continuous Renal Replacement Therapy (CRRT).** Summary of the CRRT antimicrobial dosing guide at our institution. Click here for file
