# Supplementary material for: Predictors of mortality among bacteremic patients with septic shock receiving appropriate antimicrobial therapy
Source: BMC Anesthesiol. 2014 Mar 25;14:21. doi: 10.1186/1471-2253-14-21 (PMC3987695; doi:10.1186/1471-2253-14-21)
Supplement: Additional file 1 — Elements of the sepsis resuscitation bundle. Detailed explanation of the sepsis resuscitation bundle at our institution. [file 1471-2253-14-21-S1.doc]

**Additional File 1**

**Predictors of Mortality among Bacteremic Patients with Septic Shock Receiving Appropriate Antimicrobial Therapy**

Elements of the sepsis resuscitation bundle

**Element Definition**

Lactate Measured before or within 1 hr after blood culture

Blood culture Drawn before antibiotics administered

Timely antimicrobial(s) Administered within 1 hr of sepsis recognition and intensive care unit admission

Fluid resuscitation Administration of an initial bolus of 20 mL/kg (crystalloid or equivalent colloid) administered followed by subsequent fluid challenges until one of the following:

● Central venous pressure ≥8 mm Hg (≥12 mm Hg if mechanical ventilation)

● Mean arterial pressure ≥65 mm Hg without vasopressors and lactate <2.5 mmol/L and urine output >0.5 ml/kg/hr

Appropriate vasopressor use Vasopressor administered for one of the following two:

● Persistent MAP <65 mm Hg despite fluid challenge 20 mL/kg of crystalloid

● Life-threatening hypotension with MAP <50 mm Hg for ≥15 mins

Red blood cell transfusion Transfused if hematocrit <30% and ScvO2 <70% or mixed venous O2 <65% despite fluid resuscitation

Inotrope utilization Started if Hct ≥30% and ScvO2 <70% or mixed venous oxygen saturation <65% despite fluid resuscitation
