# Supplementary material for: Predictors of mortality among bacteremic patients with septic shock receiving appropriate antimicrobial therapy
Source: BMC Anesthesiol. 2014 Mar 25;14:21. doi: 10.1186/1471-2253-14-21 (PMC3987695; doi:10.1186/1471-2253-14-21)
Supplement: Additional file 2 — Independent predictors of hospital mortality by logistic regression analysis after excluding patients transferred from an outside facility. A sensitivity analysis of the multivariable analysis whereby patients transferred from an outside facility were excluded, yielding similar results. [file 1471-2253-14-21-S2.docx]

**Additional File 2**

**Predictors of Mortality among Bacteremic Patients with Septic Shock Receiving Appropriate Antimicrobial Therapy**

Independent predictors of hospital mortality by logistic regression analysis after excluding patients transferred from an outside facility (n=193)

| **Variable** | **Adjusted Odds Ratio** | **95% Confidence Interval** | **P** |
| --- | --- | --- | --- |
| Continuous renal replacement therapy within 48 hours | 4.34 | 1.37-14.11 | 0.013 |
| Intra-abdominal source of infection | 4.69 | 1.61-14.52 | 0.005 |
| *Escherichia coli* infection | 0.21 | 0.06-0.65 | 0.006 |

Other variables included in the model with a P value of >0.05: Acute Physiology and Chronic Health Evaluation III score, vasopressor for at least 1 hour, baseline serum lactate, do not resuscitate in case of cardiac arrest status, full compliance with non-antimicrobial elements of the sepsis resuscitation bundle, median time to appropriate antimicrobial therapy, received metronidazole, enrolled after sepsis response team implementation at study ICU, urinary source of infection, *Staphylococcus aureus* infection

**74 no research authorization**

**952 no positive blood culture**

**22 did not receive appropriate**

**antimicrobial therapy within 24 hours**

**13 previously included in study**
