# Supplementary material for: Predictors of mortality among bacteremic patients with septic shock receiving appropriate antimicrobial therapy
Source: BMC Anesthesiol. 2014 Mar 25;14:21. doi: 10.1186/1471-2253-14-21 (PMC3987695; doi:10.1186/1471-2253-14-21)
Supplement: Additional file 3 — Adult Dosing for Continuous Renal Replacement Therapy (CRRT). Summary of the CRRT antimicrobial dosing guide at our institution. [file 1471-2253-14-21-S3.doc]

**Additional File 3**

**Predictors of Mortality among Bacteremic Patients with Septic Shock Receiving Appropriate Antimicrobial Therapy**

**Adult Dosing for Continuous Renal Replacement Therapy (CRRT)**

| Medication^a^ | CRRT empiric dosingb,c,d |
| --- | --- |
| anidulafungin | Unlikely to be affected by CRRT due to high protein binding and fairly large Vd  Use usual dose^e^ |
| caspofungin | Unlikely to be affected by CRRT due to high protein binding  Use usual dose but adjust for hepatic dysfunction if pertinent^e^ |
| cefazolin | 1-2 g q12h^e^ |
| cefepime | Consider 2 g q12h for life-threatening infections or intermediately susceptible organisms  1 g q12h should be adequate for susceptible organisms |
| ceftazidime | 1-2 g q12h |
| ceftriaxone | Use usual dose  1-2 g q24h for non-CNS infections or 2 g q12h for CNS infections |
| ciprofloxacin | 400 mg q12-24h |
| dalfopristin/quinupristin | Use usual dose; adjust for hepatic dysfunction if pertinent  7.5 mg/kg q8-12h |
| daptomycin | Unlikely to be affected by CRRT  Use usual dose for renal failure (4-6 mg/kg q48h) with close monitoring^e^ |
| fluconazole | Use about double the daily dose with CRRT compared with usual dose in patients with normal renal function for the specific infection type |
| ganciclovir | Maintenance: 2.5 mg/kg q24h  Induction: 2.5 mg/kg q12h (also consider 5 mg/kg q24h)^e^ |
| gentamicin | Use conventional dosing (1-2.5 mg/kg), depending on type of infection, with initial dosing interval of about q24h  Monitor serum levels and adjust dose accordingly  No data for pulse dosing |
| itraconazole | Use usual IV dose  Not affected significantly by CRRT, which appears to remove vehicle |
| levofloxacin | 500-mg load, then 250 mg q24h  Consider 500 mg q24h for severe or nosocomial infections when targeting levels similar to 750 mg q24h in healthy patients^e^ |
| linezolid | Use usual dose of 600 mg q12h  Studied with lower flow rates of 1.5-3 L/h; consider increase to 800 mg q12h or 600 mg q8h with higher flow rates or more resistant organisms |
| meropenem | 1 g q8-12h |
| metronidazole | 500 mg q6-8h^e^  Adjust dose for hepatic dysfunction if pertinent |
| micafungin | Unlikely to be affected by CRRT due to high protein binding  Use usual dose^e^ |
| moxifloxacin | Use usual dose of 400 mg q24h^e^ |
| penicillin | Consider about 6 million units per day (comparable to 20 million units when renal function is normal)^e^ |
| piperacillin/tazobactam | 2.25-3.375 g q6h^e^ or 4.5 g q8h^e^ |
| posaconazole | Unlikely to be affected due to large Vd, high protein binding, and low serum levels^e^  Use usual dose |
| tigecycline | Unlikely to be affected^e^  Use usual doses of 100-mg load, then 50 mg q12h, with close monitoring |
| trimethoprim-sulfamethoxazole  (tmp/smx) | Consider 5 mg/kg tmp q12h (comparable to about 15 mg/kg q24h tmp when renal function is normal)^e^  Monitor serum levels and adjust dose accordingly |
| vancomycin | 15-20 mg/kg q24-48h is reasonable empiric therapy Monitor serum levels and adjust dose accordingly |
| voriconazole | Use usual dose but adjust for hepatic dysfunction if pertinent^e^  CRRT appears to remove vehicle |

^a^ For drugs not included, even in the absence of good studies, equations can be used to make predictions about how they might be affected by CRRT.
^b^ CRRT flow rates affect the clearance of drugs removed by this modality. If lower flow rates are used, doses of drugs that are removed may need to be decreased. For considerably higher flow rates, doses may need to be increased.
^c^ Dosing recommendations apply to total CRRT flow rates of 3-4 L/h. Other forms of continuous replacement therapy (eg, SLED) or use of higher or lower flow rates may have different dosing needs.
^d^ Assuming minimal residual renal function, normal liver function, and total flow rates of 3-4 L/h.
^e^ Support in the medical literature is unavailable or limited; check levels when possible to confirm dose.
